# Relevance of micrometastases detected by reverse transcriptasepolymerase chain reaction for melanoma recurrence: systematic review and meta-analysis

**DOI:** 10.1590/S1516-31802003000100006

**Published:** 2003-01-02

**Authors:** Allisson Monteiro da Silva, Renato Santos de Oliveira, Lydia Masako Ferreira, Humberto Saconato

**Keywords:** Reverse transcriptase, Polymerase chain reaction, Sentinel node, Melanoma, Micrometastasis, Immunohistochemistry, Reação de polimerase em cadeia, Linfonodo, Sentinela, Melanoma, Micrometástases, Imunohistoquímica

## Abstract

**CONTEXT:**

Cutaneous melanoma presents significant morbidity and mortality. Nowadays, about 90% of them are diagnosed by clinical examination and most are localized melanomas. Sentinel node biopsy has brought about a new and interesting approach towards localized cutaneous melanoma. The meaning of micrometastases in sentinel nodes diagnosed by the reverse transcriptase-polymerase chain reaction is not well established.

**OBJECTIVE:**

To define the real value of micrometastases diagnosed by the reverse transcriptase-polymerase chain reaction in relation to melanoma recurrence.

**METHODS:**

Systematic literature review and meta-analysis. The Cochrane Library, Medline, Embase and Lilacs were the databases searched. We used the following key words: sentinel node and melanoma; sentinel node and reverse transcriptase-polymer-ase chain reaction; melanoma and reverse transcriptase-polymerase chain reaction. Cohort studies enrolling localized cutaneous melanoma patients who underwent sentinel node biopsy were selected. Sentinel node evaluations included hematoxylin and eosin, immunohistochemistry and reverse transcriptase-polymerase chain reaction.

**RESULTS:**

Out of the 1,542 studies evaluated, four were eligible. The four studies, when combined, were statistically homogeneous. The sample totaled 450 patients grouped as follows: 163 with a sentinel node negative to hematoxylin eosin and immunohistochemistry and positive to the reverse transcriptase-polymerase chain reaction; 192 with a sentinel node negative to hematoxylin eosin, immunohistochemistry and the reverse transcriptasepolymerase chain reaction and 95 patients with a sentinel node positive to hematoxylin eosin and/or immunohistochemistry. We analyzed the first two groups. The meta-analysis for the random model showed an increased effect from a positive reverse transcriptase-polymerase chain reaction on the recurrence rate. A similar result occurred in the meta-analysis for the fixed effect model.

**CONCLUSION:**

Patients with a positive reverse transcriptase-polymerase chain reaction had a greater recurrence rate than those with a negative reverse transcriptase-polymerase chain reaction. This suggests an important role for the reverse transcriptasepolymerase chain reaction in sentinel node examinations. In view of the small sample, a clinical trial could better evaluate this question.

## RESUMO

**CONTEXTO:**

O melanoma cutâneo apresenta importante morbidade e mortalidade. A incidência de melanoma cutâneo vem aumentando em todo o mundo nos últimos 50 anos. Atualmente, cerca de 90% dos casos são diagnosticados pelo exame clínico e, na maioria das vezes, ainda na forma de melanoma localizado. A biópsia de linfonodo sentinela trouxe uma nova e interessante abordagem para o melanoma cutâneo localizado.

**OBJETIVO:**

Definir o real valor de micrometástases diagnosticadas em linfonodo sentinela através da técnica de transcriptase reversa associada à reação de polimerização em cadeia (reverse transcriptase-polymerase chain reaction, PCR) na recorrência do melanoma cutâneo.

**MÉTODOS:**

Realizamos uma revisão sistemática da literatura com metanálise. Cochrane Library, Medline, Embase e Lilacs foram os bancos de dados pesquisados. Foram usadas as seguintes palavras-chave: *sentinel node and melanoma, sentinel node and reverse transcriptase-polymerase chain reaction, melanoma and reverse transcriptase-polymerase chain reaction*. Fez-se seleção de estudos prognósticos coorte, abrangendo pacientes portadores de melanoma cutâneo submetidos a biópsia de linfonodo sentinela. O exame do linfonodo sentinela incluiu análise histopatológica por hematoxilina e eosina, imunohistoquímica e reação de polimerase em cadeia. Foi realizada metanálise através do *software* RevMan (Review Manager 4.1-c; Cochrane Collaboration) com avaliação de *odds ratio* e risco relativo, utilizando modelo de efeito fixo e randômico.

**RESULTADOS:**

Entre 1.542 estudos identificados, quatro foram elegíveis para a revisão sistemática. Os quatro estudos combinados foram estatisticamente homogêneos (c2 = 2,66, p = 0,44 para *odds ratio* e c2 = 3,17, p = 0,37 para risco relativo). A casuística totalizou 450 pacientes. Esses pacientes foram distribuídos em três grupos: 163 com linfonodo sentinela negativo para hematoxilina-eosina e imunohistoquímica, sendo a reação de polimerase em cadeia positiva; 192 com linfonodo sentinela negativo para hematoxilina-eosina, imunohistoquímica e a reação de polimerase em cadeia e 95 com linfonodo sentinela positivo para hematoxilinaeosina e ou imunohistoquímica. Apenas os dois primeiros grupos foram objetos de nossa análise. A metanálise utilizando modelo de efeito randômico mostrou aumento de recorrência para o grupo com a reação de polimerase em cadeia positiva (*odds ratio* = 3,0 [1.16, 7.78] e risco relativo = 2.55 [1.06, 6.12]). Resultado similar foi encontrado na metanálise utilizando modelo de efeito fixo (*odds ratio* = 3,64 [1.51, 8.76] e risco relativo = 3.16 [1.39, 7.19]).

**CONCLUSÃO:**

Pacientes com reação de polimerase em cadeia positiva tiveram uma taxa de recorrência maior que aqueles com reação de polimerase em cadeia negativa. Esse resultado sugere um importante papel da reação de polimerase em cadeia no exame do linfonodo sentinela. Considerando-se a pequena casuística, um estudo clínico prospectivo poderia avaliar melhor esta questão.

## INTRODUCTION

Cutaneous melanoma presents significant morbidity and mortality. Its incidence has been increasing all over the world over the last 50 years. Nowadays, about 90% of them are diagnosed by clinical examination and most of them are localized melanomas.^1^ Thus, more attention has been paid to this kind of patient.

Sentinel node biopsy has brought about a new and interesting approach towards localized cutaneous melanoma.^2^ The sentinel node represents the pathological status of the lymphatic basin. Currently, it is known that the sentinel node status is the most important prognostic factor for localized cutaneous melanoma.^3^ The detection of micrometastases at sentinel nodes improves the accuracy of staging, and provides valuable prognostic information for guiding subsequent treatment.^4-7^

In the beginning, sentinel node evaluations was done via hematoxylin and eosin on serial sections. Later on, immunohistochemical studies (HMB-45 and S-100 protein) were added, improving the diagnosis of micrometastases.^8^ Recently, methods based on the reverse transcriptase-polymerase chain reaction have been used for diagnosing micrometastases.^9,10^

It is believed that micrometastases detected by the reverse transcriptase-polymerase chain reaction are really metastases with biological significance.^11-13^ However, the relevance of micrometastases detected by the reverse transcriptase-polymerase chain reaction in relation to melanoma recurrence is not well established yet. To address this interesting question, we systematically reviewed the literature and performed a meta-analysis.

## METHODS

We used the following key words for our systematic literature review: melanoma and sentinel node; melanoma and reverse transcriptasepolymerase chain reaction; and sentinel node and reverse transcriptase-polymerase chain reaction. The Cochrane Library, Medline, Embase and Lilacs were the databases used. We hand-searched all the bibliographies of the reports, papers and textbooks that we reviewed, without limiting dates or languages.

We selected cohort studies that evaluated patients with localized cutaneous melanoma who underwent sentinel node biopsy. Studies were initially selected by the title and abstract information, and then submitted to two reviewers. Sentinel node biopsies were performed using preoperative lymphoscintigraphy, intraoperative gamma probe detection and/or lymphatic mapping. The inclusion criteria can be seen in Table 1. Two groups were compared: a) patients with a sentinel node negative to hematoxylin and eosin, immunohistochemistry and reverse transcriptase-polymerase chain reaction; b) patients with a sentinel node negative to hematoxylin and eosin and immunohisto-chemistry, but positive to the reverse transcriptase-polymerase chain reaction. The main variable focused on was melanoma recurrence. The relative risk was calculated using the Review Manager (RevMan) software, considering not only random effects^14^ but also fixed effect models.^15-18^

**Table 1:** Inclusion criteria used for the systematic review

CRITERIA	DESCRIPTION
Study design	Cohort (prognostic study)
Sample	No limit
Objective	To access the recurrence rate of cutaneous melanoma among patients undergoing sentinel node biopsy
Patients	Presented localized cutaneous melanoma undergoing sentinel node biopsy
Interventions made	Preoperative lymphoscintigraphy, Intraoperative lymphatic mapping, Intraoperative gamma detection, Sentinel node biopsy, Sentinel node examination, hematoxylin and eosin, immunohistochemistry and reverse transcriptase polymerase chain reaction
Duration of follow-up (mean or median)	More than 12 months
Lost to follow-up	Less than 10%
Statistical methods	Descriptive statistics with total number and percentage of recurrence

## RESULTS

The studies selected for the systematic literature review are described in Table 2. The electronic search identified 1,542 references in all databases. Fifteen studies met the inclusion criteria. Eleven studies were excluded because the follow-up was less than 12 months or there was insufficient data on the recurrence rate. Thus, four studies were eligible.^19-22^ When the four studies were pooled, it could be demonstrated that there was statistical homogeneity in terms of the effect (X^2^ = 3.17, p = 0.37) (confidence interval = 95%). The sample size of the four included studies totaled 450 patients. These patients were grouped as follows: 163 had negative hematoxylin and eosin, negative immunohistochemistry and a positive reverse transcriptase-polymerase chain reaction; 192 had negative hematoxylin and eosin, negative immunohistochemistry and a negative reverse transcriptase-polymerase chain reaction and 95 patients had a positive sentinel node upon pathological examination (hematoxylin and eosin and/or immunohistochemistry). Only the first two groups were objects of our analysis.

**Table 2:** Final selection from the systematic literature review

Study	Patients	HE and/or IHC positive Recurrence/Total	HE and IHC negative RT-PCR positive Recurrence/Total	HE and IHC negative RT-PCR negative Recurrence/Total
Joseph et al.^19^, 1997	29	5/11	2/8	1/10
Bostick et al.^20^, 1999	72	5/17	3/20	0/35
Blaheta et al.^21^, 2000	116	9/15	4/21	10/80
Li et al.^22^, 2000	233	18/52	11/114	1/67
**Total**	**450**	**37/95**	**20/163**	**12/192**

*HE: Hematoxylin and eosin; IHC: Immunohistochemistry; RT-PCR: Reverse transcriptase polymerase chain reaction.*

**Figure 1:**
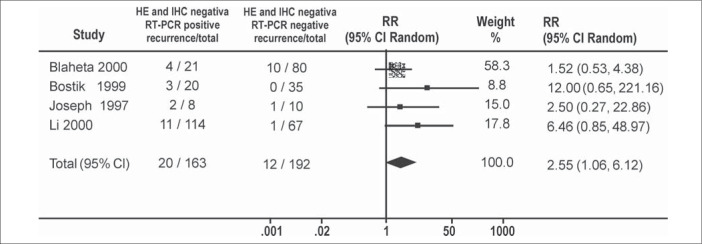
Melanoma recurrence risk due to micrometastasis diagnosed by reverse transcriptase polymerase chain reaction. Meta-analysis using random model (RR).

**Figure 2:**
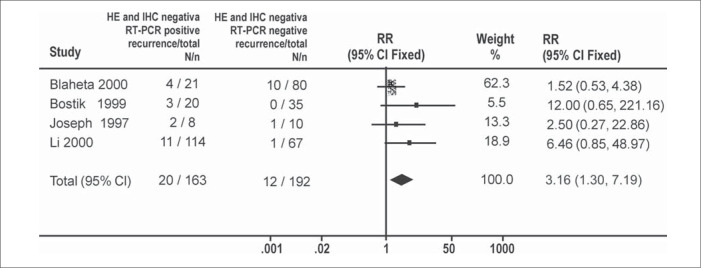
Melanoma recurrence risk due to micrometastasis diagnosed by reverse transcriptase polymerase chain reaction. Meta-analysis using fixed model (RR).

The meta-analyses for random model effect showed an increased recurrence rate caused by a positive reverse transcriptase-polymerase chain reaction (relative risk = 2.55 [1.06, 6.12]) (Figure 1). The same was observed in meta-analyses using fixed model effect (relative risk = 3.16 [1.39, 7.19]) (Figure 2).

*Test for heterogeneity: chi-squared = 3.17; degrees of freedom = 3; p = 0.37; Teste for overall effect: z = 2.09; p = 0.04. HE: Hematoxylin and eosin; IHC: Immunohistochemistry; RT-PCR: Reverse transcriptase polymerase chain reaction; CI: Confidence interval.*

*Test for heterogeneity: chi-squared = 3.17; degrees of freedom= 3; p = 0.37 Test for overall effect: z = 2.75; p = 0.006 HE: Hematoxylin and eosin; IHC: Immunohistochemistry; RT-PCR: Reverse transcriptase- polymerase chain reaction; CI - Confidence interval*

## DISCUSSION

Sentinel node biopsy allows for more accurate prognostic information for patients with localized cutaneous melanoma. The sentinel node status identifies homogeneous patient populations for clinical trials and selects patients for lymph node dissection decisions and/or adjuvant therapy (interferon alpha-2b). Optimizing the diagnosis of the micrometastasis is just as important as picking out the correct sentinel node (using an appropriate technique). Molecular techniques such as the polymerase chain reaction may be more sensitive than immunohistology, which is more sensitive than histology (hematoxylin and eosin), and the latter is more sensitive than clinical inspection.^23,24^ The fact that sentinel node positivity is the strongest prognostic factor for recurrence of localized cutaneous melanoma gives importance to micrometastases. Nevertheless, the real value of micrometastases diagnosed by the reverse transcriptase-polymerase chain reaction in relation to melanoma recurrence remains an unresolved question. That is why we conducted this systematic revision.

Molecular diagnosis of micrometastases by the reverse transcriptase-polymerase chain reaction has two advantages, at least when compared with immunohistochemistry. First, it has more sensitivity. Second, it is cheaper and quicker.^25^ Some authors question the specificity of the reverse transcriptase-polymerase chain reaction, emphasizing the risk of false positives caused by *nevi* cells, for example. This problem can be minimized using more than one mRNA (messenger ribonucleic acid) examination, like mRNA-Tyrosinase, mRNA-MART-1 (melanoma antigen recognized by T cells-1) and mRNA-MAGE III (melanoma antigen gene family 3). A multi-institutional study – the "Sunbelt Melanoma Trial" – has addressed issues regarding the relative clinical significance of microscopic nodal disease determined by histology, immunohisto-pathology and reverse transcriptase-polymer-ase chain reaction analysis, using four molecular markers, MAGE III, MART-1, GP 100 (glycoprotein 100) and Tyrosinase.^26^

In our study, there was recurrence in twelve out of the 192 reverse transcriptasepolymerase chain reaction-negative patients. Ten of them were in one single study, which had a higher percentage of patients (65%) with a Breslow thickness of more than 1.5 mm, and therefore at higher risk of nodal metastasis.

The combined studies were statistically homogenous and meta-analysis showed that melanoma recurrence was significantly influenced by the presence of micrometastases diagnosed by reverse transcriptase-polymerase chain reaction alone. Thus, the detection of micrometastases by the reverse transcriptase-polymerase chain reaction added prognostic significance to the histopathology examination. This result occurred not only when a random effect model was used but also with fixed effect models.

One limitation of this study is a possible bias in the meta-analysis due the small number of patients included in the eligible studies (450 patients). However, the concordance of the results from different analyses minimizes such limitation.

## CONCLUSION

According to our data, patients with a positive reverse transcriptase-polymerase chain reaction have more recurrence than those with a negative reverse transcriptase-polymerase chain reaction, considering all the patients with negative histology and immunopathology evaluations. This suggests an important role for the reverse transcriptase-polymerase chain reaction in sentinel node examinations. In view of the small sample, a clinical trial could better evaluate and address this question. It is suggested that minute tumor deposits from micrometastases in sentinel nodes have prognostic significance.
